# Aconitum Napellus in Neuralgia

**Published:** 1846-10

**Authors:** Alexander Fleming

**Affiliations:** Edinburgh


*Aconitum Napellus in Neuralgia.* By Alexander Fleming, of Edinburgh.—I have met with several cases of neuralgia, in which the individual had, for weeks or months, been in the habit of procuring sleep, and a temporary cessasion of pain, by opiate draughts, and who, on using the Aconite, obtained permanent relief for the disease.

Exhaustion by alcohol is the best mode of obtaining an active preparation of the plant. Cold water takes up but little of its virtues; and the expressed juice contains only a portion of its active properties; while the latter are entirely destroyed by exposure of the drug, in any of its forms, to a high temperature. These circumstances are explained by the fact, that Aconitina, the active principle of the plant, is soluble in alcohol, but not in water, and is decomposed by much heat. *Boiling in water, if long continued,* is sufficient to deprive the plant of the power of producing numbness and tingling (Geiger); but Dr. Christison has ascertained that this property is not diminished by a heat of 212°, in drying the plant, or in preparing an extract from it.

These facts account for the variable strength and frequent inertness of many of the preparations formerly in use, such as watery extracts, expressed and inspissated juices, &c.

*Tinctura Aconiti.*—Take of root of A. Napellus, carefully dried and finely powdered, sixteen ounces Troy; rectified spirit, sixteen fluid ounces; macerate for four days; then pack into percolator; add rectified spirit until twenty-four ounces of tincture are obtained.

It is beautifully transparent, of the color of sherry wine, and the taste is slightly bitter.

*Extractum Alcoholinum Aconiti.*—This is prepared by distilling, at a low temperature, the spirit from the tincture, until the consistence of an extract has been obtained. The process should be completed in a vapor bath.

Its color is dark brown, or almost black; it has an agreeable smell, and bitter taste. The dose is one-third of a grain, thrice daily, commencing with one sixth of a grain.

I prefer the tincture for external administration, from its greater uniformity of action. The method of administering it varies according to the object in view.

As an *anodyne*, *anti-neuralgic*, and *calmative*, five minims ought to be given at first, three times daily, to be increased daily to the extent of one minim each dose, until the physiological effects described under the second degree of operation have been produced.

As an *Antiphlogistic*, five minims ought to be given at first, and repeated in four hours; by which means the second degree of operation will, in all likelihood, have been induced. In order to sustain the sedative action thus developed, two and a half minims are to be given every three or four hours; or less frequently, according to the effect produced.

Where this mode of administration is adopted, it is *absolutely necessary* that the patient should be seen, and his pulse examined, before the exhibition of each dose. When this cannot be done, the remedy may be given in the manner pointed out for its use as an anodyne and calmative.

The *best method* of administering the remedy in *diseases of the heart*, is to give it in smaller doses than those recommended for its use as an anodyne, but more frequently repeated, as three or four minims fiive times daily.

Sickness may be avoided or checked by an effervescing draught, administered with, or immediately after, the dose.

The long-continued use of Aconite occasionally renders the patient less susceptible of its influence, in which case the dose must be gradually increased.

This very powerful medicine ought to be used with great caution. Like digitalis, it is cumulative in its action, and after a few days of repose exhibits itself in alarming power. In neuralgic affections it is acquiring much celebrity.

